# An Electrostatic MEMS Translational Scanner with Large Out-of-Plane Stroke for Remote Axial-Scanning in Multi-Photon Microscopy

**DOI:** 10.3390/mi8050159

**Published:** 2017-05-15

**Authors:** Haijun Li, Xiyu Duan, Gaoming Li, Kenn R. Oldham, Thomas D. Wang

**Affiliations:** 1 Department of Internal Medicine, University of Michigan, Ann Arbor, MI 48109, USA; haijunl@umich.edu (H.L.); gaomingl@umich.edu (G.L.); 2 Department of Biomedical Engineering, University of Michigan, Ann Arbor, MI 48109, USA; dxy@umich.edu; 3 Department of Mechanical Engineering, University of Michigan, Ann Arbor, MI 48109, USA; oldham@umich.edu

**Keywords:** MEMS scanner, axial scanning, multiphoton microscopy

## Abstract

We present an electrostatic microelectromechanical systems (MEMS) resonant scanner with large out-of-plane translational stroke for fast axial-scanning in a multi-photon microscope system for real-time vertical cross-sectional imaging. The scanner has a compact footprint with dimensions of 2.1 mm × 2.1 mm × 0.44 mm, and employs a novel lever-based compliant mechanism to enable large vertical displacements of a reflective mirror with slight tilt angles. Test results show that by using parametrical resonance, the scanner can provide a fast out-of-plane translational motion with ≥400 μm displacement and ≤0.14° tilt angle over a wide frequency range of ~390 Hz at ambient pressure. By employing this MEMS translational scanner and a biaxial MEMS mirror for lateral scanning, vertical cross-sectional imaging with a beam axial-scanning range of 200 μm and a frame rate of ~5–10 Hz is enabled in a remote scan multi-photon fluorescence imaging system.

## 1. Introduction

Optical imaging in vertical cross-sections with sub-cellular resolution is essential to biomedical research and clinical diagnosis because histology-like images can be provided to distinguish different features of tissue for early detection of cancer and other diseases. By combining axial and lateral scanning, this capability can be provided. Conventionally, axial scanning in confocal and multi-photon microscopes is achieved with the movement of either the objective or stage, and images in the vertical plane are reconstructed from a series of horizontal images. This approach is limited in speed and is prone to motion artifacts from vibrations introduced in the sample.

New methods to perform remote axial scanning have been developed to overcome these limitations, including group velocity dispersion (GVD)-based [1,2,3,4] and tunable lens-based temporal focusing [5,6]. Axial scanning with GVD modulation can achieve high speeds, but results in blurry images [4]. The use of a tunable lens for axial scanning has the advantages of high speed, low cost, and ease of integration, but suffers from changes in magnification and numerical aperture (NA) [6]. A remotely-located axial scan mirror that reflects the excitation beam has recently been demonstrated to move the focus [7,8,9]. High scan speeds can be achieved with mirrors that have minimal inertia. This method has been used successfully to collect aberration-free images at high speeds with a multi-photon microscope. Two high numerical aperture objectives are used to introduce equal but opposite aberrations in the excitation wavefront during scanning [8]. However, the scanning mechanisms used to move the mirror are based on bulky actuators, such as either a galvanometer or voice coil motor, which results in the difficulty of miniaturizing the system.

To realize axial scanning in a miniature instrument, a compact translational actuator that provides fast scan with large displacements and small tilting angles is needed. Microelectromechanical systems (MEMS) technology is well-suited to this application, and some recent advances have been made in MEMS translational actuators. In general, to achieve large displacements, thermoelectric, piezoelectric, and electromagnetic actuation mechanisms are usually used in MEMS actuators. Thermoelectric actuators can provide large displacements at low voltages, but have slow response times [10,11]. Thin-film piezoelectric scanners can achieve large displacements with high speeds, but require complex fabrication processes [12,13]. Electromagnetic actuators have been developed with fast response times and good displacement, but this technology has high power consumption and is difficult to scale down in size [14,15]. Compared with other actuation mechanisms, electrostatic approaches typically have small actuation force and the pull-in effect, but offer the advantages of low power consumption and complementary metal-oxide-semiconductor (CMOS)-compatible fabrication. Electrostatic MEMS actuators that use the principle of parametric resonance have achieved large axial displacements up to several hundreds of microns [16,17,18,19].

We have previously demonstrated an electrostatic MEMS scanner with axial scan capabilities to collect vertical cross-sectional images in a dual-axis confocal endomicroscope [17] and a multi-photon microscope [19]. Here we demonstrate a MEMS scanner with a smaller footprint and a higher speed to further extend the applicability of this axial scan technique in a multi-photon microscope system for real-time vertical cross-sectional imaging.

## 2. Scanner Design and Fabrication

### 2.1. MEMS-Based Remote-Scan Multi-Photon Imaging System

**Figure 1:**
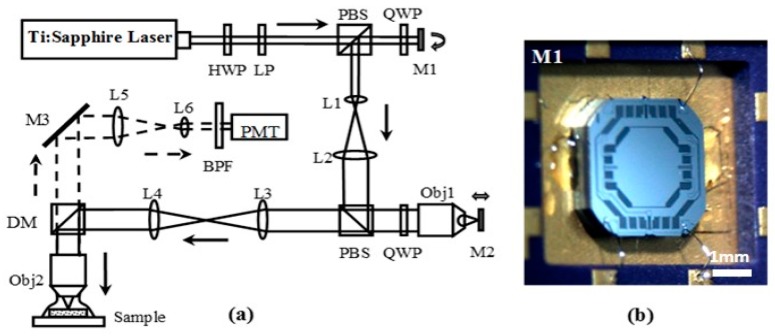
(**a**) Schematic for microelectromechanical systems (MEMS)-based remote scan multi-photon microscopic system. Key: HWP: half wave plate, LP: linear polarizer, L1-6: lenses, Obj1-2: objectives, M1: MEMS bi-axial torsional mirror for lateral scanning, M2: MEMS out-of-plane translational scanner, M3: fixed reflective mirror, PBS: polarizing beam splitter, QWP: quarter wave plate, DM: dichroic mirror, BPF: band pass filter, PMT: photomultiplier tube; (**b**) photo of MEMS bi-axial torsional mirror.

Figure 1a shows the schematic of a MEMS-based remote scan multi-photon imaging system. A bi-axial torsion MEMS mirror (M1, Figure 1b) and an out-of-plane translational MEMS scanner (M2) are used to perform lateral and axial scanning, respectively. Figure 1b shows the bi-axial torsional MEMS mirror [20]. This scanner employs a gimbal geometry that enables a 1.8 mm-diameter reflective mirror to rotate around the inner *X*- and outer *Y*-axes. The *X*-axis is defined as the fast axis with a resonant frequency of ~4.3 kHz, and the *Y*-axis is defined as the slow axis with a resonant frequency of ~1.05 kHz. Based on the optical design of the remote scan unit, the translation of M2 results in the axial displacement of the focus below the tissue surface with a magnification of ~2:1. That is, to achieve an imaging depth of 200 μm, the axial scanning device needs to provide a displacement of 400 μm.

### 2.2. Design of the Out-of-Plane Translation MEMS Scanner

**Figure 2:**
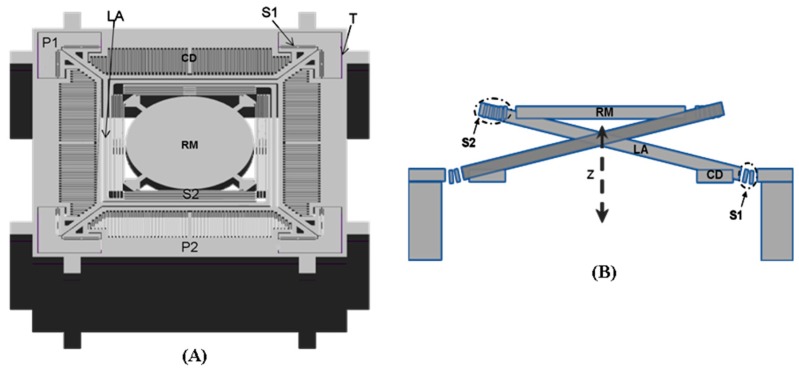
Schematics for out-of-plane translational MEMS scanner with a level-based compliant mechanism. (**A**) Front view of the basic structure; (**B**) cross-section view of the out-of-plane translational motion enabled by the level-based compliant mechanism. Key: CD: comb drive, LA: spiral-like level arm, P1-2: electrical pads, RM: reflective mirror, S1: H-shaped torsional spring, S2: multi-turn folded-beam spring, T: electrical isolation trench.

The basic structure for the out-of-plane translational MEMS device used for axial scanning in the multi-photon imaging instrument is shown in Figure 2A. A central reflective mirror is supported by four lever-based suspensions, and four comb-drives are used for actuation. The suspensions and the mirror form a compliant mechanism that can transfer the rotation of the lever into vertical translation of the mirror. This device is fabricated in a silicon on insulator (SOI) wafer with movable structures, comb-drives, and electrical pads formed in the silicon device layer. A cavity is opened in the silicon handle layer, and narrow trenches are opened in the silicon device layer for electrical isolation. The scanner has a dimension of 2.1 mm × 2.1 mm × 0.54 mm for integration into a miniature instrument. The mirror is designed with a diameter of 0.8 mm to cover the focused beam dimension over the expected 400 μm scan range. The lever-arm of the suspension is designed to have a spiral shape with a length of 1.33 mm to achieve a large vertical displacement. It also couples to the mirror and the anchor through two H-shaped torsional springs and one multi-turn folded-beam spring, respectively. The design of the H-shaped torsional spring is used to enable large rotations while providing high resistance to lateral bending, and the design of the multi-turn folded-beam spring is used to enable large deflections in its folding direction while providing a high resistance to lateral bending. The comb-drive has an in-plane structure, in which movable and stationary comb fingers with the same thickness are formed in the silicon device layer. Unlike conventional vertical staggered comb-drives, the in-plane comb-drive can only work in resonance to enable out-of-plane motions of the mirror. Based on the principle of parametric resonance, a driving voltage signal with a frequency near at 2 ω_0_/*n*—where ω_0_ is the natural frequency of the out-of-plane motion and *n* is an integer ≥1—should be used for actuation.

**Figure 3:**
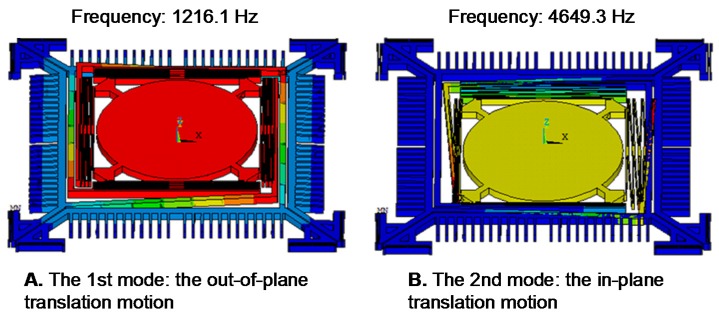
Finite element method (FEM) modal analysis. (**A**) The first mode (1216 Hz): the desired out-of-plane translational motion; (**B**) the second mode (4649.3 Hz): the in-plane translation motion.

**Figure 4:**
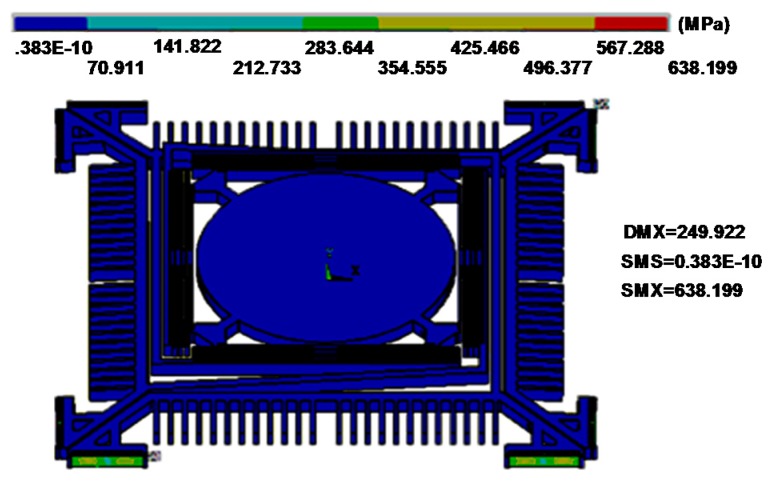
Stress distribution with a ±250 µm out-of-plane vertical displacement. Maximum stress of ~638.2 MPa is found near the fixed end of the H-shaped torsional spring.

We optimized the geometry of springs to provide fast stable axial scanning with >400 μm displacement while avoiding spring failure. Figure 3 shows results for modal analysis of the optimized scanner using ANSYS software. The first mode is chosen as the desired out-of-plane translational motion with a resonant frequency of 1216.1 Hz, and the second mode is an in-plane translational motion with a resonant frequency of 4649.3 Hz. According to these results, the optimized structure will provide a stable out-of-plane translational motion with high resistance to parasitic vibrations. Figure 4 shows the stress distribution through the scanner, where there is a maximum value of ~638.2 MPa near at the fixed end of the H-shaped torsional spring with ±250 μm axial displacement. This value is well below the limit for fracture strength of single crystal silicon [21].

### 2.3. Fabrication Process

**Figure 5:**
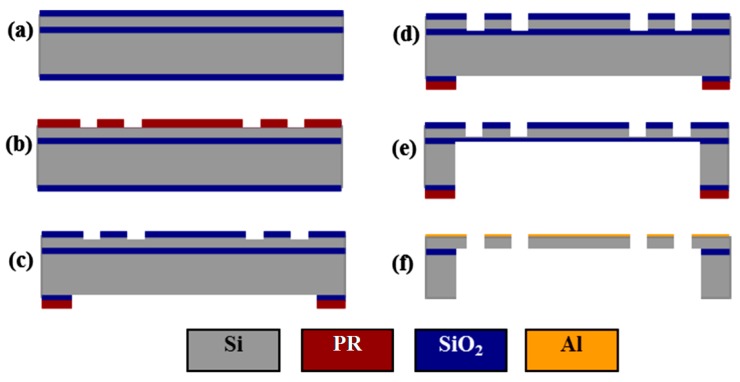
Process flow: (**a**) deposition of plasma-enhanced chemical vapor deposition (PECVD) SiO_2_ hardmask layers; (**b**) patterning of the front side SiO_2_ layer; (**c**) patterning of the backside SiO_2_ layer; (**d**) deep reactive-ion etching (DRIE) of the device layer; (**e**) DRIE of the handle layer; (**f**) SiO_2_ buffered hydrofluoric acid solution (BHF) release-etching, isopropyl alcohol (IPA) rinsing, IPA drying, and evaporation of Al layer.

A robust SOI micromachining process is developed for fabrication, which achieves a yield of >95%. Figure 5 shows the process flow, which starts with a 4-inch SOI wafer with a 40 μm silicon device layer, a 1 μm silicon dioxide (SiO_2_) buried layer, and a 500 μm silicon handle layer. To avoid scratching and contaminating the reflective mirror surface, a 0.5 μm SiO_2_ film was used as a hard mask, and was first deposited on the surface of the device layer by a plasma-enhanced chemical vapor deposition (PECVD) process. A 1 μm PECVD SiO_2_ layer was also deposited on the backside surface to avoid photoresist burning during the deep reactive-ion etching (DRIE) process for removing backside silicon with a large open area (Figure 5a). Two masks (Figure 5b,c) and two DRIE silicon etching steps (Figure 5d,e) were used to define and form the scanner structures in the device and handle layers of the SOI wafer. The movable structures were released using a buffered hydrofluoric acid solution (BHF) to etch away the SiO_2_ layers followed by an isopropyl alcohol (IPA) rinsing and drying. A 70 nm layer of aluminum (Al) film was coated on the device layer to provide >85% reflectivity over the visible and near-infrared spectrum (Figure 5f). This film was also used as the metal contact layer for electrical pads to perform wire bonding.

## 3. Performance Characterization

**Figure 6:**
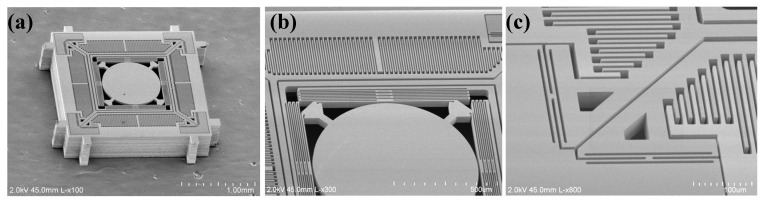
Scanning electron microscope (SEM) images of the fabricated scanner. (**a**) the complete device structure; (**b**) the multi-turn folded-beam spring; and (**c**) the H-shaped torsional spring.

Figure 6 shows scanning electron microscope (SEM) images of a fabricated device. The surface quality of the reflective mirror was characterized by an optical surface profiler (NewView 5000, Zygo, Berwyn, PA, USA). Measurements show that the mirror has a radius of curvature of ~2.6 m and a root mean square (RMS) roughness of ~2 nm.

**Figure 7:**
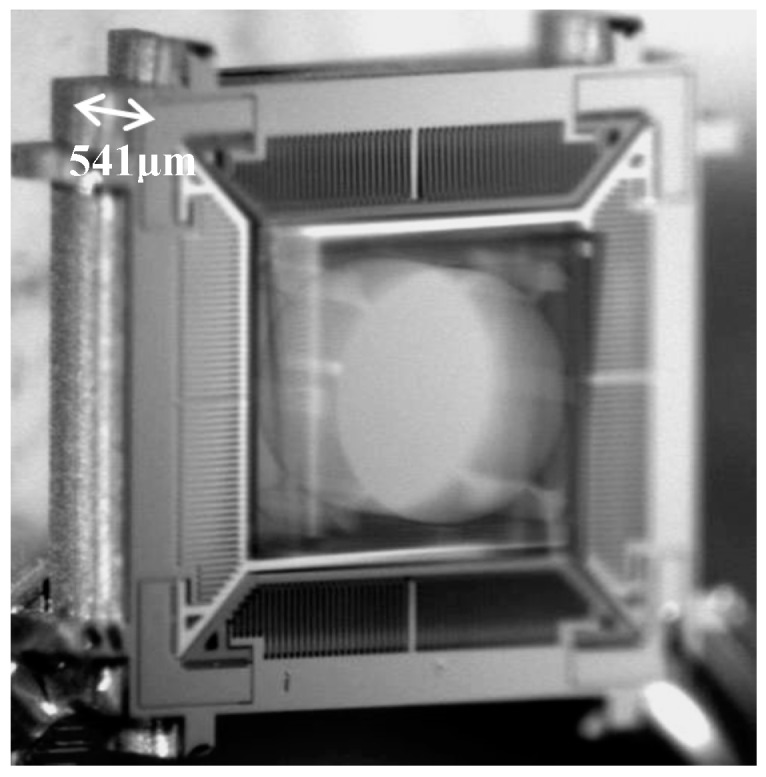
Blur motion image of the scanner in out-of-plane translational resonant mode.

We characterized the dynamic performance of the scanner using a displacement sensor to measure the out-of-plane translational displacement and a position sensing detector (PSD) to determine the tilt angle. Due to the compact geometry of the air damping, the squeeze film effect especially has a significant impact on out-of-plane translation. We reduced damping and achieved high-amplitude out-of-plane translation under ambient conditions by mounting the scanner onto a substrate with a ~0.5 mm-deep open-wall cavity. The scanner was driven into resonance by sweeping the drive frequency of a square-wave voltage at near twice the natural frequency of the out-of-plane translational mode. Figure 7 shows an image of the out-of-plane blur motion of the scanner.

**Figure 8:**
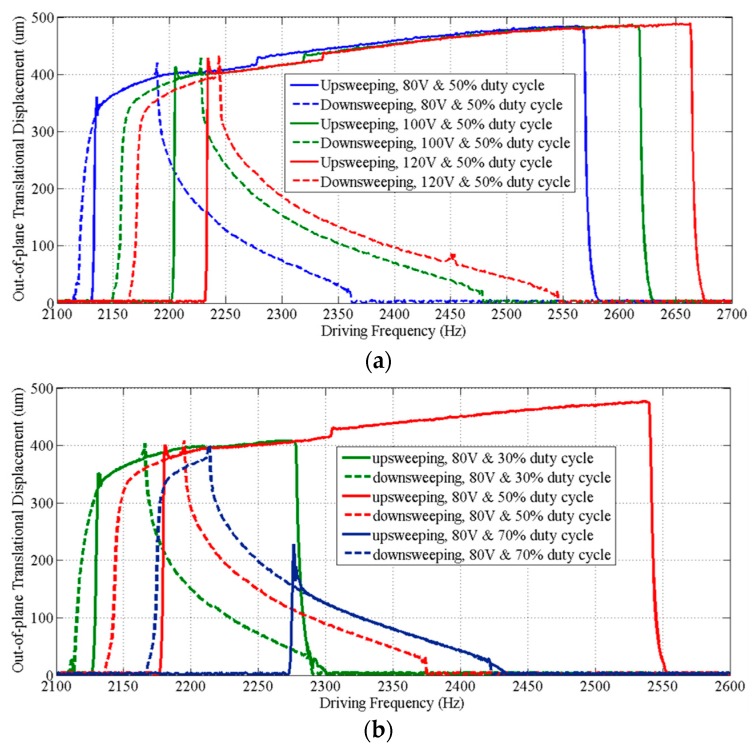
Frequency response curves of the out-of-plane translation motion. (**a**) Driven by square-wave signals with a 50% duty cycle at various voltages, the scanner exhibits a stiffness hardening behavior and a stiffness softening–hardening-mixed behavior during frequency upsweep and downsweep of the drive signals, respectively; (**b**) driven with square-wave signals that have different duty cycles at 80 V, the scanner exhibits not only stiffness hardening or softening–hardening-mixed behaviors, but also a stiffness softening behavior with upsweep of the frequency of a drive signal with a 75% duty cycle.

**Figure 9:**
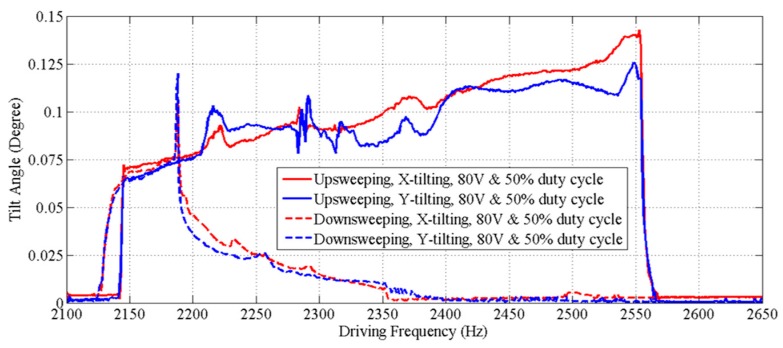
Frequency response curves for tilt angles over the frequency response range of the out-of-plane translational motion. These curves are similar to those for translation, and have the same response frequency range.

Figure 8 shows that the dynamic response curves of the scanner exhibit a complex dynamic nonlinearity. We observed stiffness softening, mixed softening–hardening and hardening behaviors in the device by adjusting the voltage (Figure 8a) and the duty cycle (Figure 8b) of the square-wave drive signal. A relatively flat response region with large amplitudes (>400 µm) and wide adjustable frequency range (~390 Hz) was observed when forward sweeping the frequency of a drive signal with 80 V and 50% duty cycle, and a maximum amplitude of 480 µm was obtained at ~2.57 kHz. Measurements of the tilt angles about *X* and *Y* axes of the mirror over the frequency range for out-of-plane translational motion are also shown (Figure 9). The tilt angle is ≤0.14° for both *X* and *Y* axes when driven by a drive signal with 80 V and 50% duty cycle. The response curves for tilting and translation are similar and have the same frequency response range. This result suggests that tilting is not from vibration in other mechanical modes, but rather caused by process variations in the geometry of individual springs, resulting in the asymmetry of the scanner.

## 4. Imaging Result

**Figure 10:**
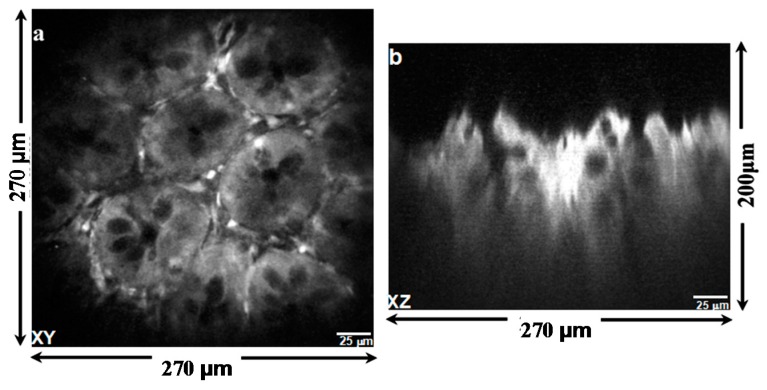
Multi-photon excited fluorescence images of mouse colonic epithelium that constitutively expresses tdTomato ex vivo reveal crypt structure. (**a**) The image in horizontal (XY) plane was collected using the biaxial MEMS scanner only; (**b**) the image in vertical (XZ) plane collected by using both the lateral and axial MEMS scanners.

Multi-photon excited fluorescence images of mouse colonic epithelium that express tdTomato were collected ex vivo at a frame rate of 5 Hz. Figure 10a shows a representative image obtained in the horizontal (XY) plane over a field of view (FOV) of 270 µm × 270 µm. For horizontal cross-sectional images, only the biaxial MEMS scanner was used to perform 2D Lissajous scanning in the XY plane. The inner *X*-axis and the outer *Y*-axis were respectively defined as the fast axis and the slow axis. Figure 10b shows an image of the same specimen obtained in the vertical (XZ) plane over a FOV of 270 µm × 200 µm by using the biaxial MEMS scanner and the out-of-plane translation scanner to perform 1D lateral scanning and axial-scanning, respectively.

## 5. Discussion and Conclusions

Axial scanning is needed to collect optical sections of tissue in the vertical plane, the direction for development of normal epithelium and invasion of disease. Standard objectives or stages that move in this dimension are slow, and collected images are prone to movement artifacts. Using a remotely located axial scanner/mirror is a promising technique that overcomes many limitations of conventional methods. A light-weight compact mirror that performs fast axial scanning may improve performance and extend applicability of this technique to miniature imaging instruments. This work presents a novel compact MEMS out-of-plane translational scanner developed to perform fast axial-scanning for a multi-photon microscopic system with a remote scan architecture. This scanner can achieve a fast (~1.27 kHz) out-of-plane translational motion with large axial displacements (≥400 μm) and slight tilt angles (≤0.14°) at ambient pressure. By employing this scanner and a biaxial MEMS mirror, vertical cross-sectional imaging with a beam axial-scanning range of 200 μm and a frame rate of ~5–10 Hz are enabled. The ability to acquire 3D images is limited because the scanner works in resonant mode only. Optical magnification in the current multi-photon system is ~2:1. This can introduce sensitivity to optical aberrations and difficulty for scanner design. The lever-based compliant mechanism demonstrated in this work can quasi-statically transfer small tilt angles into large pure axial displacements if vertically staggered comb drives are used. Future work will further optimize the structural design and modify the fabrication process to develop a scanner that can work in quasi-static mode.

## References

[1] Durst M.E., Zhu G., Xu C. (2006). Simultaneous spatial and temporal focusing for axial scanning. Opt. Express.

[2] Du R., Bi K., Zeng S., Li D., Xue S., Luo Q. (2008). Analysis of fast axial scanning scheme using temporal focusing with acousto-optic deflectors. J. Mod. Opt..

[3] Straub A., Durst M.E., Xu C. (2011). High speed multiphoton axial scanning through an optical fiber in a remotely scanned temporal focusing setup. Biomed. Opt. Express.

[4] Dana H., Shoham S. (2012). Remotely scanned multiphoton temporal focusing by axial grism scanning. Opt. Lett..

[5] Grewe B.F., Voigt F.F., Hoff M.V., Helmchen F. (2011). Fast two-layer two-photon imaging of neuronal cell populations using an electrically tunable lens. Biomed. Opt. Express.

[6] Jiang J., Zhang D., Walker S., Gu C., Ke Y., Yung W.H., Chen S.-C. (2015). Fast 3-D temporal focusing microscopy using an electrically tunable lens. Opt. Express.

[7] Botcherby E.J., Juskaitis R., Booth M.J., Wilson T. (2007). Aberration-free optical refocusing in high numerical aperture microscopy. Opt. Lett..

[8] Botcherby E.J., Smith C.W., Kohl M.M., Débarre D., Booth M.J., Juškaitis R., Paulsen O., Wilson T. (2012). Aberration-free three-dimensional multiphoton imaging of neuronal activity at kHz rates. Proc. Natl. Acad. Sci. USA.

[9] Rupprecht P., Prendergast A., Wyart C., Friedrich R.W. (2016). Remote z-scanning with a macroscopic voice coil motor for fast 3D multiphoton laser scanning microscopy. Biomed. Opt. Express.

[10] Wu L., Xie H. (2008). A large vertical displacement electrothermal bimorph microactuator with very small lateral shift. Sens. Actuators A Phys..

[11] Zhang X., Zhou L., Xie H. (2015). A fast, large-stroke electrothermal MEMS mirror based on Cu/W bimorph. Micromachines.

[12] Qiu Z., Pulskamp J., Lin X., Rhee C., Wang T., Polcawich R., Oldham K. (2010). Large displacement vertical translational actuator based on piezoelectric thin-films. J. Micromech. Microeng..

[13] Zhu Y., Liu W., Jia K., Liao W., Xie H. (2011). A piezoelectric unimorph actuator based tip-tilt-piston micromirror with high fill factor and small tilt and lateral shift. Sens. Actuators A Phys..

[14] Mansoor H., Zeng H., Chen K., Yu Y., Zhao J., Chiao M. (2011). Vertical optical sectioning using a magnetically driven confocal microscanner aimed for in vivo clinical imaging. Opt. Express.

[15] Zeng H., Chiao M. (2007). Magnetically actuated MEMS microlens scanner for in vivo medical imaging. Opt. Express.

[16] Sandner T., Grasshoff T., Gaumont E., Schenk H., Kenda A. (2014). Translatory MOEMS actuator and system integration for miniaturized Fourier transform spectrometers. J. Micro Nanolithogr. MEMS MOEMS.

[17] Li H., Duan X., Qiu Z., Zhou Q., Kurabayashi K., Oldham K.R., Wang T.D. (2016). Integrated monolithic 3D MEMS scanner for switchable real time vertical/horizontal cross-sectional imaging. Opt. Express.

[18] Li H., Duan X., Wang T.D. An electrostatic MEMS scanner with in-plane and out-of-plane two-dimensional scanning capability for confocal endoscopic in vivo imaging. Proceedings of the 2017 IEEE 30th International Conference on Micro Electro Mechanical Systems (MEMS).

[19] Duan X., Li H., Li X., Oldham K.R., Wang T.D. (2017). Axial beam scanning in multiphoton microscopy with MEMS-based actuator. Opt. Express.

[20] Duan X., Li H., Zhou J., Zhou Q., Oldham K.R., Wang T.D. (2016). Visualizing epithelial expression of EGFR in vivo with distal scanning side-viewing confocal endomicroscope. Sci. Rep..

[21] Chen K.S., Ayon A., Spearing S.M. (2000). Controlling and testing the fracture strength of silicon on the mesoscale. J. Am. Ceram. Soc..

